# Medical News

**Published:** 1856-10

**Authors:** 


### THE MEDICAL EXAMINER.

PHILADELPHIA, OCTOBER, 1856.

### MEDICAL NEWS.

MASSACHUSETTS STATE MEDICAL SOCIETY.—*Prize Question for 1857.*—The Massachusetts Medical Society is authorized, by a donation from one of its members, to offer the sum of *One Hundred Dollars* for the best Dissertation adjudged worthy of a prize on the following theme, viz :—

" *We would regard every approach towards the rational and successful prevention and management of disease, without the necessity of drugs, to be an advance in favor of humanity and scientific medicine.*"

The packet accompanying the successful Dissertation will be broken in open meeting, and the author's name announced, at the annual meeting of the Society in May, 1857.

Dissertations for the above prize must be sent (post-paid), to the Corresponding Secretary, Dr. C. E. WARE, No. 6 Temple Place, Boston, on or before April 15th, 1857.

By order of the Councillors,

B. E. COTTING, *Rec. Sec'y.*

*Roxbury, Nov.* 1, 1855.

---

Dr. Cock has been chosen President of the College of Physicians and Surgeons of New York, in the place of Dr. Stevens, resigned. Professor Le Conte has resigned the Chair of Chemistry, and Dr. Samuel St. John has been elected to the vacancy.

---

Dr. Timothy Childs, of Pittsfield, Mass., has accepted the Chair of Anatomy in the New York Medical College, previously held by Dr. E. H. Parker.

---

A case of drowning, successfully treated according to Dr. Marshall Hall's plan, (see Sept. No. of the Examiner,) is related in the August No. of the London Lancet. The patient is stated to have not been less than from fifteen to twenty minutes completely submerged after he had risen to the surface for the last time."

As it may interest some of our readers to know where copies of the works published by the Sydenham Society may be obtained, we copy the following from a circular sent us :—

The Sydenham Socety, of London.—List of the Society's Works, of which copies are still on hand, and from which new members, subscribing for the current year, may make a selection, on payment of an additional five dollars for any three volumes, with the exception of those to which an asterisk is affixed. Those to which an asterisk is affixed, or any other single volume, may be had for $2 50 per volume.

Sydenhami Opera Omnia. 1 vol.
Hasse's Pathological Anatomy. 1 vol.
Rhazes on the Small-pox and Measles. 1 vol.
The Works of Hewson. Portrait and plates. 1 vol.
Dupuytren's Lectures on Diseases and Injuries of Bones. 1 vol.
Dupuytren on Lesions of the Vascular System, &c. 1 vol.
Memoirs of the French Academy of Surgery. 1 vol.
Feuchtersleben's Medical Psychology. 1 vol.
Microscopical Researches of Schwann and Schleiden. 1 vol. Plates.
The Works of W. Harvey, M. D. 1 vol.
The Genuine works of Hippocrates. 2 vols.
Essays on Puerperal Fever, and other Diseases Peculiar to Women. 1 vol.
The Works of Sydenham, translated from the Latin. 2 vols.
Unzer and Prochaska on the Nervous System. 1 vol.
Annals of Influenza. 1 vol.
Romberg on Diseases of the Nervous System. 2 vols.
Kolliker's Manual of Human Histology. 2 vols. Woodcuts.
*Rokitansky's Pathological Anatomy. Complete in 4 vols.
*Hunter on the Gravid Uterus. 1 vol. folio. 34 plates, with descriptive letter-press.
Wedl's Pathological Histology. 1 vol. Woodcuts.
Oesterlen's Medical Logic. 1 vol.
Velpeau on Diseases of the Breast. 1 vol.
The Works of Aretæus, Greek and English. 1 vol.

The subscription, constituting a member, is five dollars annually, payable in advance.

Three volumes, handsomely bound in a uniform manner in cloth, gilt edged, are usually issued in the year.

Richard J. Dunglison, M. D.,
*Hon. Local Secretary for Philadelphia.*

—

The Provincial Medical Associatin has accomplished, very quietly, its transformation into the British Medical Association—a change, the very proposal of which, last year, threatened the disruption of the body, and met with such decided opposition, that not a few of its members felt it to be their duty to resign connection with a Society of such limited aspirations. We are glad, however, to find that a spirit of harmony now exists in the ranks of this very important body, and we trust that its present prosperous condition will be turned to good account, and that its influence will be used in promoting the best interest of the profession, apart from petty sectional rivalries. The "Association Journal" is, of course, to participate in the change of name, and will speedily be issued as the "British Medical Journal." Its existence has given rise to much discussion from time to time, and we have always been of opinion that the funds of the Association might be more usefully employed than in the maintenance of such an expensive periodical. We must, however, do the present Editor the justice to observe, that he has successfully guided its fortunes in a very perilous juncture, and that he continues to make good use of the materials placed at his disposal. By the laws of the Association, the appointment of Editor is vested in the Committee of Council. To this, objection was made, and we think justly,—several members protesting against the responsibility of electing so important an officer of the Association being left in the hands of half a dozen persons. An amendment, which proposed that the Editor should be elected by the whole Council, was, unfortunately, lost.—*Edinburgh Medical Journal,* Sept., 1856.

---

THE USE OF GLYCERINE FOR THE PRESERVATION OF ORGANIC BODIES.—Luton states that animal and vegetable substances may be kept for a long period perfectly free from decomposition when immersed in glycerine. He also finds that it is a good antiseptic agent for injecting dead bodies.—*London Lancet.*

---

POISONING BY CHLOROFORM.—The most extraordinary overdose of chloroform yet known was wilfully swallowed by a patient recently in London. The man drank about four ounces at one draught! Wild intoxication, followed by profound insensibility, ensued; but, after various relapses and accidents, he is now quite well.—*Lon.don Lancet.*

---

REMARKABLE CASE.—Mrs. Julia Syles, wife of John Syles, of Blackstone, died on the 14th ult. of dropsy, from which she had suffered for five years. During that time she had been tapped upwards of one hundred and forty times, and more than three thousand pounds of water were extracted!—*London Lancet.*

—

International Banquet to Crimean Surgeons in Paris.—On Wednesday evening, August 27, a memorable international dinner was given in the splendid dining hall of the Hotel du Louvre by the Medical profession of Paris to the French, English, Sardinian, and Turkish surgeons who served in the Crimean war, when upward of 600 people sat down to dinner. Baron Paul Dubois, the senior member of the Faculty of Medicine, and the accoucheur who attended the Empress at her confinement, took the chair. At the desert, Baron Duboise proposed the following toasts:—"The names of the medical men who died in the East," and "The Emperor of the French." Several other toasts were drank all bearing upon the medical services rendered in the late war. One of them was proposed by Sir J. Hall, C. B. A toast was proposed to the Russian doctors who had humanely taken care of our prisoners.—*Edinburg Med. Jour.*

—

Air-Poison.—People have often said that no difference can be detected in the analyzation of pure and impure air. This is one of the vulgar errors difficult to dislodge from the public brain. The fact is, that the condensed air of a crowded room gives a deposit which, if allowed to remain for a few days, forms a solid, thick, glutinous mass, having a strong odour of animal matter. If examined by the microscope, it is seen to undergo a remarkable change. First of all, it is converted into a vegetable growth, and this is followed by the production of multitudes of animalcules; a decisive proof that it must contain organic matter, otherwise it could not nourish organic beings. This was the result arrived at by Dr. Angus Smith, in his beautiful experiments on the Air and Water of Towns; wherein he showed how the lungs and skin gave out organic matter, which is in itself a deadly poison, producing headache, sickness, disease, or epidemic, according to its strength. Why, if "a few drops of the liquid matter, obtained by the condensation of the air of a foul locality, introduced into the vein of a dog, can produce death with the usual phenomena of typhus fever," what incalculable evil must not it produce on those human beings who breathe it again and again, rendered fouler and less capable of sustaining life with each breath drawn? Such contamination of the air, and consequent hot-bed of fever and epidemic, it is easily within the power of man to remove. Ventilation and cleanliness will do all, so far as the abolition of this evil goes, and ventilation and cleanliness are not miracles to be prayed for, but certain results of common obedience to the laws of God.—*Dickens' Household Words, from Edin. Med. Jour.*

---

Golden Days of the Apothecaries.—When physician and apothecary were good friends, and the physician was a man who, in the phrase of the trade—for here we must needs call it a trade—could write well, something like this was the result. We quote only one day's medicine, prescribed by a physician, and administered by an apothecary, to a fever patient. The list of medicine given on each other day is quite as long, and every bolus is found in the same way duly specified in "Mr. Parret the apothecary's bill, sent in to Mr. A Dalley, who was a mercer on Ludgate Hill." We quote the supply for the fourth day's illness:—

| | *August* 10. | | |
|---|---|---|---|
| Another Pearl Julap . . . . | £0 | 6 | 10 |
| Another Hypnotick Draught . . | 0 | 2 | 0 |
| A Cordial Bolus . . . . | 0 | 2 | 0 |
| A Cordial Draught . . . . . | 0 | 1 | 8 |
| A Cordial Pearly Emulsion . . . | 0 | 4 | 6 |
| Another Pearl Julap . . . | 0 | 6 | 8 |
| Another Cordial Julap . . . | 0 | 3 | 8 |
| Another Bolus . . . . . | 0 | 2 | 4 |
| Another Draught . . . . . | 0 | 1 | 8 |
| A Pearl Julap . . . . . | 0 | 4 | 6 |
| A Cordial Draught . . . . | 0 | 2 | 0 |
| An Anodyne Mixture . . . . | 0 | 4 | 6 |
| A Glass of Cordial Spirits . . . | 0 | 2 | 0 |
| Another Mucilage . . . . . | 0 | 3 | 4 |
| A Cooling Mixture . . . . | 0 | 3 | 6 |
| A Blistering Plaister to the Neck . . | 0 | 2 | 6 |
| Two more of the same to the Arms . | 0 | 5 | 0 |
| Another Apozem . . . . . | 0 | 3 | 6 |
| Spirit of Hartshorn . . . . | 0 | 0 | 6 |
| Plaister to dress the Blisters . . | 0 | 0 | 6 |

One day's medical treatment is here represented, as it was often to be met with in the palmy days of physic, when

Some fell by laudanum, and some by steel,
And death in ambush lay in ev'ry pill.

Then truly might Dr. Garth write of his neighbors, how

The piercing caustics ply their spiteful pow'r,
Emetics wrench, and keen cathartics scour.
The deadly drugs in double doses fly;
And pestles peal a martial symphony.

—*Edinburgh Med. Journ. from Household Words.*

